# Exploring Service Use and Patients' Pathways in a Community Neuropsychiatry Clinic, a Service Evaluation: What Does the Data Tell Us?

**DOI:** 10.1192/bjo.2026.11639

**Published:** 2026-06-30

**Authors:** Olubunmi Olure, Asmaa Hussein, Obumneme Chinweuba, Verity Williams, Samr Dawood

**Affiliations:** Kent and Medway Mental Health NHS Trust, Kent, United Kingdom

## Abstract

**Aims:**

We aimed to explore the characteristics of referrals to an NHS outpatient neuropsychiatry service, define patient pathways, and identify interventions provided.

**Methods:**

We included all new referrals to the service between 1/10/2024 and 31/01/2025. We examined electronic clinic records (Rio) retrospectively and extracted anonymised data using an NHS-approved online data-collection tool (‘Gather’). We used ‘Gather’ and Excel for data analysis, employing descriptive statistics. 165 records were reviewed.

**Results:**

93% of referred patients were of working age, with 4 referrals aged under 18.Most were female (64%) and 80% were white British. 129 patients had working status recorded: 52% received welfare benefits. 75% had social support; mostly from family. Most referrals came from Primary Care and Neurology.

70% of referrals had a functional neurological disorder (FND) (30% non-epileptic attack disorder (NEAD), 40% other FND). Other diagnoses included epilepsy (n=8), traumatic brain injury (n=1), Huntington’s disease (n=2), MS (n=2) and Tourette's/tic disorder (n=4). 103 patients (62%) had previous psychological history and 59% had previous mental health service contact. 67% were prescribed psychotropic medications at referral. The most common comorbid mental health problem was anxiety (45 patients, 27%), 37 patients had depression (22%). 16 patients were autistic (10%). 70% had previous physical health problems; 39% previous neurological problems, 18% non-neurological problems and 43% both.

All referrals received multidisciplinary team (MDT) review; 52% of referrals were accepted following this. Mean waiting time between received referral and MDT discussion was 10 days. Most patients (52%) had documented investigations at referral. Of these, 81% had brain imaging or EEG completed. Mean waiting time for initial assessment/triage was 52 days; most patients were triaged by specialist nurse. 14% of referrals were discharged post-triage, mostly to primary care/self-help. Average length of stay within the service was 233 days. During the period evaluated, 19% of patients were receiving psychotherapy (11% 1:1 therapy, 8% group). 16 patients were referred to neurophysiology. 36% were prescribed medication during their treatment, mostly antidepressants. Of discharged patients, 67% were discharged to GP and 15% to community mental health services.

**Conclusion:**

Referrals to the community neuropsychiatry service were characterised by complex comorbidities and clinical challenges. A significant percentage of referrals involved functional neurological presentations, with psychiatric comorbidities and social vulnerabilities. Clients received multiple types of interventions. The findings emphasise the importance of multidisciplinary assessments and integrated care pathways to address the needs of this population and guide future service development.

Additional authors: Dr Sylvia Fatunla, Prof Rafey Faruqui

